# Effect of Almond Milk Versus Cow Milk on Postprandial Glycemia, Lipidemia, and Gastrointestinal Hormones in Patients with Overweight or Obesity and Type 2 Diabetes: A Randomized Controlled Clinical Trial

**DOI:** 10.3390/nu17132092

**Published:** 2025-06-24

**Authors:** Shilton Dhaver, Marwa Al-Badri, Joanna Mitri, Abd Almasih Barbar Askar, Adham Mottalib, Osama Hamdy

**Affiliations:** 1 Clinical, Behavioral & Outcomes Research, Joslin Diabetes Center, Boston, MA 02215, USA; 2 Department of Medicine, Harvard Medical School, Boston, MA 02115, USA

**Keywords:** dairy, almond milk, free fatty acids, type 2 diabetes, obesity

## Abstract

Background: Almond milk is often seen as a healthier alternative to cow milk. However, its effect on postprandial glycemia compared to 2% milk is unclear. Here, we compared the postprandial glycemic effect of almond milk versus carbohydrate- or caloric-matched 2% milk, each served with oatmeal to patients with type 2 diabetes (T2D). Methods: In this crossover, three-way, open-label study, 22 participants (mean age 66 ± 7.4 years, 36% female), with T2D and overweight or obesity, consumed oatmeal served with almond milk (ALM), carbohydrate-matched 2% milk (MLKCRB), or calorie-matched 2% milk (MLKCAL) on separate days and in a random order. The primary outcome was glucose incremental area under the curve for 240 min (iAUC0-240). The secondary outcomes included postprandial serum insulin, glucagon, plasma free fatty acids (FFAs), serum triglycerides, leptin, and gastrointestinal hormones (PYY, active GLP-1, GIP, amylin, cholecystokinin, and ghrelin). Results: We did not find any difference in either the primary endpoint or secondary endpoints between the three groups. However, iAUC0-240 for insulin and glucagon was significantly higher in MLKCRB vs. ALM (FDR = 0.002 and 0.02, respectively). Conclusions: Almond milk does not offer any additional glycemic benefit over 2% milk and does not differ in its postprandial effects on FFAs, serum triglycerides, leptin, and gastrointestinal hormones over 4 h. Nonetheless, carbohydrate-matched 2% milk elicited greater insulin and glucagon response compared to almond milk, warranting further investigation into its long-term implications.

## 1. Introduction

Type 2 diabetes (T2D) is a chronic metabolic condition that has become a major global health issue, impacting millions of people worldwide [1]. Dietary modification remains a cornerstone of T2D management, with increasing emphasis on optimizing macronutrient quality and the glycemic impact of commonly consumed foods. Among these, breakfast composition is especially critical, as it can influence postprandial glycemic excursions, hormonal responses, and satiety signaling throughout the day. Oatmeal, a widely consumed carbohydrate-rich breakfast option, is known to help reduce postprandial plasma glucose levels and is often recommended for individuals with diabetes [2].

In the past few years, plant-based milk alternatives, like almond milk, have gained popularity as healthier substitutes for traditional cow milk due to their lower caloric and fat content. This shift is driven by the presumed association between dietary fat, particularly saturated fat, and cardiovascular and metabolic diseases. However, the metabolic effects of almond milk in people with diabetes remain unclear [3,4]. Dietary guidelines have increasingly recommended reducing fat and increasing carbohydrates to lower cardiovascular risk, but this shift has also been associated with a rising prevalence of obesity and T2D [5,6]. This paradox makes us question how dietary changes really affect metabolic health and how different dietary components that are frequently consumed impact blood glucose levels. Additionally, the presumed superiority of almond milk in diabetes diets is largely anecdotal and has not been thoroughly examined in controlled postprandial studies. Most prior research has focused on chronic almond consumption (e.g., as whole nuts or in the context of mixed diets), while few have investigated almond milk specifically or its acute effects on postprandial metabolism, insulin, glucagon, and gastrointestinal hormones. Furthermore, previous studies have not adequately explored how almond milk compares to carbohydrate- or calorie-matched cow milk when consumed with high-glycemic index foods such as oatmeal.

We previously demonstrated that increased dairy consumption, irrespective of fat content, over a 24-week period to ≥3 servings/day compared to <3 servings/day, while maintaining the same energy intake, did not significantly impact key metabolic parameters, such as A1C, body weight, lipid profiles, or blood pressure, in patients with T2D [7]. These findings suggest that factors other than fat content might influence the metabolic effects of dairy products. Given the increasing popularity of milk alternatives and the ongoing debate about the metabolic implications of dairy consumption, a comprehensive study comparing the effects of almond milk versus cow milk on postprandial glycemia in individuals with T2D is needed.

This study aims to evaluate the 4 h postprandial glycemic effect of almond milk (ALM) compared to 2% milk, either matched for caloric content (MLKCAL) or matched for carbohydrate content (MLKCRB), when consumed with a similar amount of oatmeal. This study also aims to investigate the differences between the three groups in postprandial serum insulin, serum glucagon, plasma free fatty acids (FFAs), serum triglycerides, serum leptin, and serum gastrointestinal hormones: polypeptide YY (PYY), active glucagon-like peptide-1 (GLP-1), glucose-dependent insulinotropic peptide (GIP), amylin, cholecystokinin, and ghrelin in individuals with overweight or obesity and T2D.

## 2. Methods

### 2.1. Study Design and Subjects

This is a prospective crossover, three-way, open-label clinical study conducted at Joslin Diabetes Center in Boston, MA. The institution’s Committee on Human Studies approved the study protocol (2016–22) on 18 September 2019, and the participants signed its informed consent form prior to enrollment. The study was conducted according to the ethical standards established by the Declaration of Helsinki for research involving human participants.

**Figure 1:**
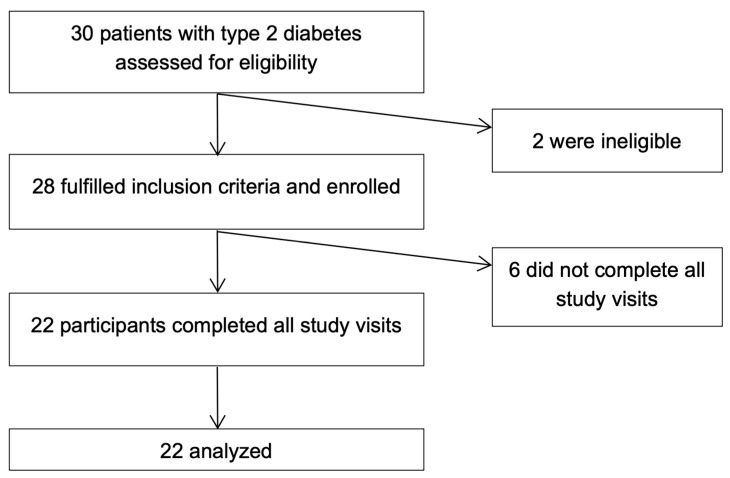
Flow diagram of study enrollment.

The inclusion criteria were ages 18 to 75, type 2 diabetes for at least three months prior to screening, and BMI > 25 kg/m^2^. The exclusion criteria were pregnancy or lactation, treatment with insulin, lactose intolerance, cow milk or nut allergies, history of bariatric surgery, and chronic gastrointestinal conditions such as gastroparesis, Crohn’s disease, ulcerative colitis, celiac disease, and irritable bowel disease. Thirty participants were assessed for eligibility, twenty-eight of whom were eligible and enrolled in the study. Subsequently, 22 participants completed all study visits and were included in the final analysis (Figure 1).

### 2.2. Study Procedures

Each eligible participant was scheduled for three separate study visits over a 6-week period, with a minimum washout interval of 3 days between any two visits to minimize potential carryover effects from prior test meals. This duration was chosen to allow adequate scheduling flexibility for participants, reduce participant burden, and ensure sufficient metabolic washout between visits. The 6-week timeframe also allowed all visits to be completed without altering participants’ usual medication regimens or introducing seasonal dietary variation, thereby improving the internal validity and consistency of the data collected. Changes in antihyperglycemic medications were not allowed during that period.

The participants were asked to fast overnight prior to each of their study visits. They were instructed to hold their antihyperglycemic medications on the morning of the visit, and to take the medication after the last blood sample was drawn. The participants treated with DPP-4 inhibitors were asked to hold their dose on the day before the test and on the morning of the test.

Anthropometric measurements and venous blood samples were collected at each visit by a qualified study team member in accordance with standardized protocols. Body weight was measured using a calibrated digital scale (Tanita BWB–800). Height was measured without shoes using a stadiometer.

Subsequently, each participant was asked to consume an oatmeal breakfast that contained ½ cup (40 g) of oatmeal that was randomly served with one of the following: (a) 360 mL almond milk (1.5 cups), (b) 246 mL 2% milk (1 cup), or (c) 182 mL 2% milk (0.74 cups). The macronutrient contents of each meal are summarized in Table 1. The oatmeal was prepared by adding 8 fl oz (237 mL) of water to 40 g (½ cup) of dry oats (Quaker Old Fashioned Oats, Quaker Oats Co., Chicago, IL, USA) then cooking it on a stove for 5–10 min. After allowing it to cool, it was served along with the milk. No sugar or flavoring was added to the meal. The participants had 5 min to consume the meal.

### 2.3. Sample Collection and Processing

Venous blood samples were drawn using an indwelling catheter immediately before meal consumption and at 15, 30, 60, 90, 120, 180, and 240 min postprandially. Fasting samples were collected into two tubes: a BD Vacutainer^®^ K2 EDTA 7.2 mg tube and a BD Vacutainer^®^ P800 plasma tube (BD, Franklin Lakes, NJ, USA). Prior to blood transfer, 50 µL of AEBSF protease inhibitor was added to the P800 tube using a ½ cc insulin syringe (1 unit = 10 µL), ensuring a final ratio of 10 µL of inhibitor per 1 mL of blood. Five milliliters of collected blood were then transferred to the P800 tube, and the remaining volume was transferred to the EDTA tube. Both tubes were gently inverted 5–10 times to ensure proper mixing with their respective additives.

Following collection, both tubes were centrifuged at 1000× *g* for 10 min at 4 °C. Plasma was carefully aspirated and aliquoted into pre-labeled cryogenic vials corresponding to each analyte and time point. Plasma from the EDTA tubes was used for analysis of free fatty acids, while plasma from the P800 tubes was used for metabolic hormone measurements. Cryovials were transferred to storage boxes and placed in a −80 °C freezer for future batch analysis.

### 2.4. Sample Analysis

Blood samples were tested for plasma glucose, serum insulin, serum glucagon, plasma FFAs, serum triglycerides, serum leptin, and serum gastrointestinal hormones: PYY, active GLP-1, GIP, amylin, cholecystokinin, and ghrelin. Plasma FFAs were determined and processed as described by Baylin et al. [8] and Zock et al. [9,10]. Hormone concentrations were determined by Luminex bead-based multiplex immunoassay, ELISA, and enzymatic method assay platforms which tested samples in singlicate with automated liquid handling (AssayGate, Inc., Ijamsville, MD, USA).

### 2.5. Statistical Analysis

Descriptive statistics were used to summarize the demographic and clinical characteristics of the study participants and expressed as mean ± standard deviation (SD) or as a mean (95% confidence interval).

Analysis was performed using repeated measures analysis of variance with treatment and time as fixed effects and subject as random effect. Incremental area under the curve between 0 and 240 min (iAUC_0–240_) was calculated for each variable using the trapezoid formula. The Wilcoxon signed rank test was used to calculate *p*-values, with *p* < 0.05 used to determine statistically significant differences between groups. *p*-values for analytes other than glucose were adjusted using false discovery rate (FDR), where FDR < 0.05 was used to determine significance.

## 3. Results

In this study, we evaluated 22 participants with T2D with a mean age of 66 ± 7.4 years, 36% females, mean diabetes duration of 11 ± 4.9 years, and mean BMI of 30 ± 3.4 kg/m² (Table 2).

**Figure 2:**
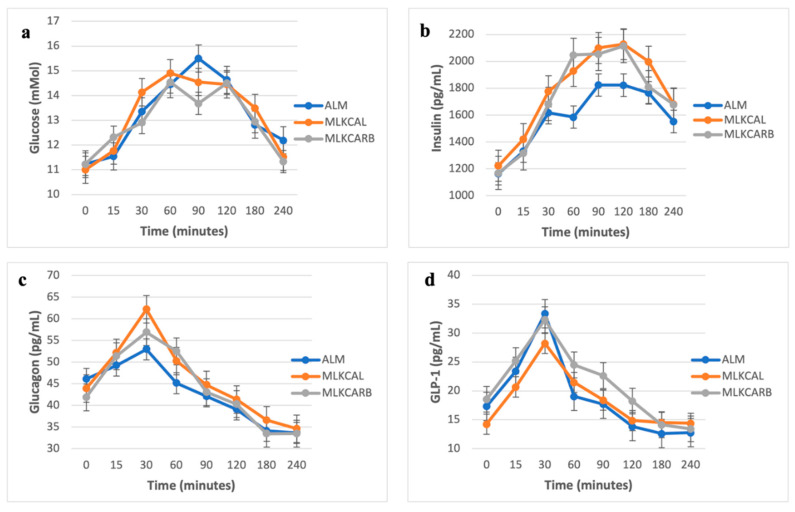
Postprandial glucose (**a**), insulin (**b**), glucagon (**c**), and GLP-1 (**d**) levels in response to the breakfast meals. Values are mean ± SEM.

**Figure 3:**
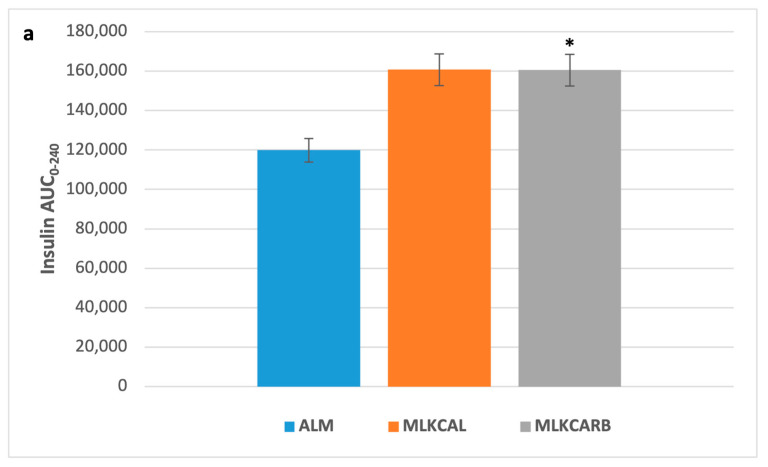

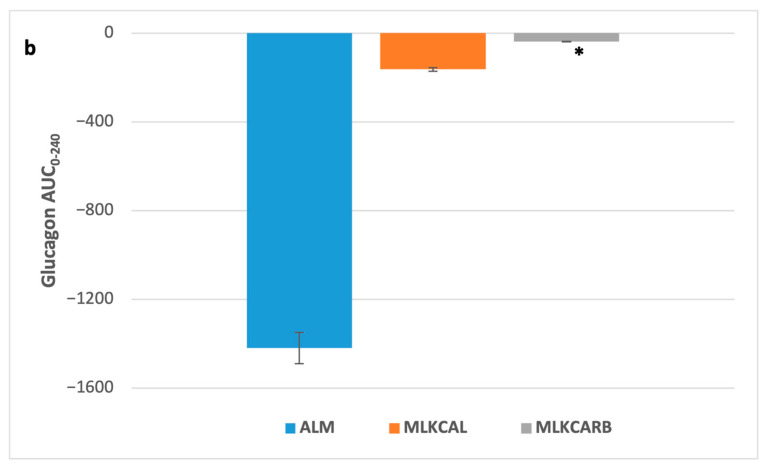
Postprandial insulin (**a**) and glucagon (**b**) incremental area under the curve 0–240 min in response to the breakfast meals. Values are mean ± SEM. * FDR < 0.05 compared to almond milk.

There were no significant differences in all the measured parameters between the three groups at baseline (Figure 2). In pair-wise comparisons, there were also no differences in postprandial iAUC_0-240_ of plasma glucose and all other measured parameters between the three groups, except for serum insulin and serum glucagon, which were significantly higher in MLKCARB versus ALM (FDR = 0.002 and FDR = 0.02, respectively) (Table 3 and Figure 3).

## 4. Discussion

In this randomized controlled clinical trial, we investigated the postprandial effects of almond milk compared to carbohydrate- or calorie-matched 2% milk in individuals with overweight or obesity and T2D. Our findings revealed that the consumption of almond milk with oatmeal did not result in any differences in postprandial plasma glucose, serum triglycerides, plasma FFAs, serum leptin, or serum levels of several gastrointestinal hormones when compared to calorie- or carbohydrate-matched 2% milk over 240 min.

Interestingly, we observed a notable elevation in insulin and glucagon secretion among the participants who consumed carbohydrate-matched 2% milk in comparison to almond milk. Under normal physiology, insulin suppresses glucagon secretion through paracrine signaling within the pancreatic islets. However, in individuals with T2D, this mechanism is often impaired. In this study, although insulin increased after consumption of 2% milk, glucagon also increased, suggesting that the expected suppression may be blunted in T2D, or that the stimulatory effects of protein and fat override insulin’s inhibitory effect on glucagon [11]. This aligns with the concept of impaired islet cell crosstalk in T2D. Milk proteins are known to provide a rich source of amino acids, particularly branched-chain amino acids like leucine, isoleucine, and valine, which directly and indirectly trigger pancreatic beta cells to release insulin [12]. This insulinogenic response is further supported by the enhancement of incretin hormone secretion (such as GLP-1 and GIP), which helps amplify the insulin release following nutrient ingestion [13,14]. At the same time, these amino acids also stimulate the pancreatic alpha cells to release glucagon. This dual hormone response is crucial and acts in concert to ensure that blood glucose levels remain stable, preventing hypoglycemia that might occur from a sudden insulin spike. While the insulinotropic effects of milk have been more extensively studied, the glucagon response, though typically less pronounced, plays a protective counter-regulatory role [15]. Fat, however, mainly plays a modulatory role by slowing gastric emptying and the subsequent absorption of other nutrients. This delay helps temper rapid spikes in blood glucose and the hormone responses that follow, rather than directly stimulating the pancreatic beta or alpha cells. Almond milk, on the other hand, does not significantly stimulate insulin secretion because it lacks the carbohydrates and proteins that typically trigger an insulin response [16,17]. This suggests that almond milk has a minimal effect on postprandial insulin secretion.

Our observation implies that the protein and fat contents of cow milk may play a role in influencing pancreatic hormonal secretion of insulin and glucagon. These findings are consistent with a previous observation that protein and fat intake elicit a stronger insulin response compared to carbohydrate intake in individuals with T2D, highlighting how the macronutrient composition of a meal can influence insulin and glucagon secretion, thereby impacting glycemic control [18,19,20].

The lack of significant differences in postprandial serum triglyceride between the almond milk group and the 2% milk group is consistent with previous observations comparing plant-based milk alternatives to cow milk [21,22,23]. Similar to 2% milk, almond milk is low in saturated fat and cholesterol, which are known to influence postprandial lipids. This may explain why we did not observe significant differences in serum triglyceride responses between the study groups. Furthermore, our study evaluated the effects of almond milk on the serum levels of several gastrointestinal hormonal secretions, including PYY, GLP-1, GIP, amylin, cholecystokinin, and ghrelin, which are crucial in regulating appetite and maintaining postprandial plasma glucose levels. Consistent with another study, our findings indicate that the consumption of almond milk does not significantly impact the secretion of these hormones compared to cow milk, suggesting that both beverages produce similar satiety and postprandial metabolic responses [21].

Consistent with our findings, previous studies also indicated that consumption of almonds does not markedly influence postprandial glucose levels in individuals with prediabetes or T2D. For example, Wien et al. demonstrated in people with prediabetes that consuming an almond-enriched American Diabetes Association (ADA) diet did not induce significant changes in postprandial glycemia compared to consuming a standard ADA diet [24]. These findings suggest that almond milk may elicit a comparable glycemic response to cow milk when consumed alongside a carbohydrate-rich meal. Conversely, another study demonstrated that the incorporation of almonds in a well-balanced healthy diet leads to multiple beneficial effects on glycemic and cardiovascular disease risk factors in Asian Indian patients with T2D [25]. This suggests that almond milk may be a suitable substitute for cow milk in managing postprandial glycemia among individuals with type 2 diabetes in certain ethnic populations [18]; however, randomized–controlled trials are needed to validate these findings.

It is important to acknowledge the limitations of our study. First, the sample size was relatively small, which may limit the generalizability of our findings. While our crossover design helped to mitigate inter-individual variability and allowed each participant to serve as their own control, the study may have been underpowered to detect smaller or more nuanced differences between interventions. Second, the open-label nature of the study introduces the potential for bias, as the study team were not blinded to the type of milk consumed during each visit. Additionally, the carbohydrate-matching strategy used in this study did not differentiate between simple and complex carbohydrates, which may have influenced hormonal responses, particularly insulin secretion. Finally, being a cross-sectional, mixed meal study precludes any conclusions regarding the long-term effects of almond milk or 2% cow milk consumption on glycemic control and other metabolic outcomes in individuals with T2D. Longitudinal studies are warranted to evaluate whether the differences observed—particularly in insulin and glucagon secretion—translate into clinically meaningful changes over time. Despite these limitations, from the mechanistic point of view, the randomized crossover design of this study offered advantages such as reduced variability and the ability to examine temporal effects within the same participants.

## 5. Conclusions

Our study provides evidence that almond milk consumption does not differ in its postprandial glycemic effect from 2% cow milk, when matched for caloric content or carbohydrate content in patients with overweight or obesity and type 2 diabetes. Carbohydrate-matched 2% cow milk resulted in higher insulin and glucagon secretions compared to almond milk, highlighting the potential influence of milk composition on pancreatic hormonal secretion. Further longitudinal randomized–controlled studies with larger sample sizes are warranted.

## Figures and Tables

**Table 1:** Nutritional information of the three breakfast meals.

ALM: Oatmeal + Almond Milk (1.5 Cups) *	
Carbohydrates	27.3 g + 12 g = 39.3 g
Protein	5.0 g + 1.5 g = 6.5 g
Fat	3.0 g + 3.8 g = 6.8 g
Calories	150 kcal + 90 kcal = 240 kcal
MLKCRB: Oatmeal + Carbohydrate-matched 2% Milk (1 cup)	
Carbohydrates	27.3 g + 12 g = 39.3 g
Protein	5.0 g + 8.1 g = 13.1 g
Fat	3.0 g + 4.8 g = 7.8 g
Calories	150 kcal + 122 kcal = 272 kcal
MLKCAL: Oatmeal + Calorie-matched 2% Milk (0.74 cups)	
Carbohydrates	27.3 g + 8.88 g = 36.2 g
Protein	5.0 g + 6.0 g = 11.0 g
Fat	3.0 g + 3.6 g = 6.6 g
Calories	150 kcal + 90 kcal = 240 kcal

* Calculation based on Silk Original Almond Milk: https://silk.com/products/original-almondmilk (accessed on 3 September 2019).

**Table 2:** Characteristics of study participants (*n* = 22).

Gender	
Male	14 (64%)
Female	8 (36%)
Age (years)	66 ± 7.4
BMI (kg/m^2^)	30 ± 3.4
Diabetes duration (years)	11 ± 4.9
Participants using diabetes medications (*n*)	22
Participants using lipid-lowering medications (*n*)	15

Gender *n* (%). Values are mean ± standard deviation.

**Table 3:** Incremental area under the curve 0–240 min for the outcome variables in response to the three meals.

	Almond Milk (ALM)	Calorie-Matched 2% Milk (MLKCAL)	Carbohydrate-Matched 2% Milk (MLKCARB)
Glucose (mM)	550.23 ± 518.52	617.73 ± 424.53	474.40 ± 555.05
Insulin (pg/mL)	119,921.59 ± 166142.80	160,748.50 ± 214,665.20	160,581.57 ± 177,361.60 *
Glucagon (pg/mL)	−1418.86 ± 2151.29	−162.61 ± 3142.83	−37.99 ± 1849.96 *
Triglycerides (mM)	−21.14 ± 67.07	37.50 ± 82.45 *	−16.80 ± 56.04
Active Ghrelin (pg/mL)	−4180.98 ± 9148.15	−2124.87 ± 7268.45	1186.76 ± 5237.95
Total peptide YY (pg/mL)	779.24 ± 3887.09	2273.74 ± 3595.94	2823.81 ± 5459.89
Active GLP1 (pg/mL)	−117.61 ± 2968.72	760.23 ± 1524.81	415.62 ± 3810.31
Active Amylin (pg/mL)	892.24 ± 1910.84	1150.12 ± 1805.47	1054.52 ± 1493.46
Leptin (pg/mL)	−348,345.68 ± 435,386.80	−288,347.73 ± 343,471.50	−245,751.77 ± 419,293.50
GIP (pg/mL)	13,890.34 ± 13,590.38	15,117.61 ± 7477.78	16,124.42 ± 9831.71
Cholecystokinin (pg/mL)	−2896.70 ± 10,809.27	−2070.00 ± 16,739.91	−1286.06 ± 13,608.64
Palmitic Acid	37.50 ± 222.63	−67.16 ± 250.05	58.44 ± 281.24
Stearic Acid	−49.09 ± 122.16	−6.48 ± 110.69	11.79 ± 114.64
Oleic Acid	18.07 ± 182.15	−78.75 ± 234.49	−84.30 ± 154.23
Linoleic Acid	35.80 ± 150.84	14.32 ± 132.45	−28.78 ± 115.74
Arachidonic Acid	22.84 ± 138.19	29.66 ± 157.69	15.10 ± 92.94
Adrenic Acid	−14.32 ± 75.81	−2.39 ± 66.29	12.95 ± 72.35
DHA	11.59 ± 63.5	19.09 ± 93.64	26.59 ± 82.18

Values are mean ± SEM. * *p* < 0.05 or FDR < 0.05 compared to ALM. Abbreviations: GLP-1: Glucagon-like Peptide-1; GIP: Glucose-dependent Insulinotropic Peptide; DHA: Docosahexaenoic acid.

## Data Availability

The data contained in this manuscript are held at the Joslin Diabetes Center Clinical Research Center and are available upon request.
